# A behaviorally informed financial education program for the financially vulnerable: Design and effectiveness

**DOI:** 10.3389/fpsyg.2022.1090024

**Published:** 2022-12-15

**Authors:** Ernst-Jan de Bruijn, Gerrit Antonides, Tamara Madern

**Affiliations:** ^1^Department of Economics, Leiden University, Leiden, Netherlands; ^2^Department of Social Sciences, Wageningen University, Wageningen, Netherlands; ^3^Institute of Law, HU University of Applied Sciences, Utrecht, Netherlands

**Keywords:** financial literacy and education, financial behavior, financially vulnerable people, behavioral insights, quasi-experimental field study, financial education

## Abstract

Financially vulnerable consumers are often associated with suboptimal financial behaviors. Evaluated financial education programs so far show difficulties to effectively reach this target population. In our attempt to solve this problem, we built a behaviorally informed financial education program incorporating insights from both motivational and behavioral change theories. In a quasi-experimental field study among Dutch financially vulnerable people, we compared this program with both a control group and a traditional program group. In comparison with the control group, we found robust positive effects of the behaviorally informed program on financial skills and knowledge and self-reported financial behavior, but not on other outcomes. Additionally, we did not find evidence that the behaviorally informed program performed better than the traditional program. Finally, we discuss the findings and limitations of this study in light of the financial education literature and provide implications for policymaking and directions for future research.

## Introduction

A growing number of studies show the occurrence of suboptimal financial behaviors among low-income, low-educated, and unemployed consumers. These behaviors range from undersaving (Shurtleff, 2009) to excessive and overly expensive overborrowing (Skiba and Tobacman, 2008; Cole et al., 2014), as well as poor financial management (Perry and Morris, 2005; De Beckker et al., 2019). Furthermore, low-income and low-educated consumers are less able to deal with shocks in income or expenses, for example, due to the Covid-19 outbreak, which makes them financially vulnerable (Hasler et al., 2018). Survey studies consistently show that financially vulnerable consumers have lower levels of financial knowledge and skills than others (Bernheim, 1998; Lusardi and Mitchell, 2014; De Beckker et al., 2019; Klapper and Lusardi, 2020; Lusardi et al., 2020b), while lower levels of financial knowledge are associated with poorer financial management (Hilgert et al., 2003), poorer credit behavior (Lusardi and Tufano, 2015), and lower financial well-being (Yakoboski et al., 2020). Traditionally, these findings are seen as strong motivations to educate financially vulnerable consumers to foster healthy financial behavior and to improve their financial well-being.

However, the literature is not conclusive whether financial education is an effective policy program, both in general and in targeting the financially vulnerable. Literature reviews are inconclusive, either suggesting the effectiveness of financial education (e.g., Lusardi and Mitchell, 2014) or concluding the opposite (Willis, 2011). Furthermore, meta-analyses provide mixed results. Fernandes et al. (2014) report only very small effects of financial education on financial knowledge and financial behavior. More recent meta-studies show that financial education can impact both financial knowledge and financial behavior (Kaiser and Menkhoff, 2017; Kaiser et al., 2022), or at least some financial behaviors, like record keeping and savings (Miller et al., 2015). However, the effects on financial behavior are small-to-medium.^1^ Furthermore, both Fernandes et al. (2014) and Kaiser and Menkhoff (2017) found that financial education is less effective for low-income clients.^2^ This raises the question also proposed by Kaiser and Menkhoff (2017): What can be done to make financial education more effective, specifically for financially vulnerable consumers?

The purpose of our study is to investigate the impact of a behaviorally informed financial education program (BI program) targeting financially vulnerable consumers in comparison to both a control group and a traditional financial education program (traditional program). Several studies suggest that effective financial education programs require a combination of transfer of knowledge, skill-building, and increasing motivation to create the desired changes in behavior (Hilgert et al., 2003; Peeters et al., 2018). The traditional program primarily focuses on the transfer of knowledge and skill-building. In designing the BI program, we supplemented these elements of the traditional program with three elements focusing on motivation and practicing. First, the program contains a need-driven and adaptive approach in which participants decide which topics will be discussed. Second, trainers apply insights from autonomy-supportive teaching (Ryan and Deci, 2000a; Kusurkar et al., 2011), the stages-of-change model (Prochaska et al., 1992; Peeters et al., 2018), and interviewing techniques (Miller and Rollnick, 2002) to enhance intrinsic motivation, solve ambivalence, and support behavioral change. Third, the program contains implementation assignments to enhance practicing and behavioral change. To keep the extent of the program the same, the behaviorally informed program pays less attention to the transfer of knowledge in comparison with the traditional program. Our research question is: Does the BI program improve financial outcomes, both in comparison with no program and a traditional financial education program?

In collaboration with five Dutch local government and debt counseling organizations, we conducted a quasi-experimental study including financially vulnerable individuals. Using a difference-in-difference design, we compared the BI program with both a traditional program and a control group. We collected data both before the start of the program (baseline) and 6 months later (endline). We estimated the effects on five groups of outcomes (indices): financial skills and knowledge, financial-psychological indicators, financial behavior, financial well-being, and financial situation. In addition, we collected data during the last program session to gain insight into the program-related outcomes. The main finding of our study is that the BI program had a positive impact on self-reported financial skills and knowledge and financial behavior but not on financial well-being and financial situation. Also, the BI program was not more effective than the traditional program.

Our work contributes to the existing literature in two ways. First, by developing a financial education program based on behavioral insights and investigating whether such a program could be (more) effective in targeting financially vulnerable consumers. Prior studies have shown that offering the program on teachable moments (Kaiser and Menkhoff, 2017) and adjusted content and didactics (e.g., rules-of-thumb-based instructions, and activating and differentiating didactics; Drexler et al., 2014; Kaiser and Menkhoff, 2018; Nagel et al., 2019; Iterbeke et al., 2020) can improve the effectiveness of financial education programs. However, the literature lacks studies that evaluate the impact of programs building on motivational insights (Peeters et al., 2018). Second, our work focused on an important but underexamined target population, namely financially vulnerable subjects. As discussed, financially vulnerable individuals may have large potential to benefit from financial education. However, only a limited number of studies investigated the impact of classroom-based financial education for this target population (Collins, 2013; Reich and Berman, 2015).^3^ We aimed at filling these gaps.

This paper proceeds as follows. In “Design of the financial education programs,” we discuss the design of both the traditional and the behaviorally informed program. “Materials and methods” describes the quasi-experimental design and sample. In “Data and empirical strategy,” we describe the data and empirical strategy. “Results” discusses our results. “Discussion” concludes with a discussion of our findings and provides some advice for policymakers.

## Design of the financial education programs

### Design of the traditional program

In addressing suboptimal financial behaviors and outcomes, financial education programs have traditionally focused on financial literacy as primary determinant. These programs build on human capital theory applied to financial behavior (Lusardi and Mitchell, 2014; Entorf and Hou, 2018), assuming that: (1) Poor financial decisions are caused by a lack of financial skills and knowledge (lack of human capital), (2) financial education (knowledge transfer and training skills) improves the participants’ financial skills and knowledge, and (3) subsequently, participants will make better (more well-informed) financial decisions, (4) which will contribute to one’s financial well-being and financial situation. The traditional program in the Netherlands has been developed by the National Institute for Family Finance Information (Nibud) and is used by a wide range of Dutch local government and non-profit organizations.^4^ The program has been designed for financially vulnerable individuals and its purpose is to promote healthy financial behaviors. The traditional program follows the rationale of human capital theory and focuses on transfer of knowledge and training skills.

Table 1 provides a summary of the characteristics of the traditional program. The traditional program has a method-driven approach with a fixed set-up including a fixed order of topics. The program uses standard course materials including a workbook for participants and a manual for trainers.^5^ The program comprises eight elementary training modules and some additional modules. The elementary modules focus on essential financial knowledge (e.g., distinguishing types of income, expenditures, and financial products) and skills (e.g., budgeting, applying for additional allowances, and keeping track). Importantly, the program pays limited attention to psychological aspects such as motivation and self-regulation. In the first session, participants set their own goal for the program by completing a statement (“I want to achieve the following with the course…”). During the last session, participants make an action plan and receive tips to stick to it. The program is meant for a small group-based or classroom setting including a trainer and 8–15 participants. The program consists of five-to-six weekly sessions of 2.5 h resulting in a total of 12.5–15 h of financial education.^6^

**Table 1:** Summary of the financial education programs.

	Traditional program	Behaviorally informed program
*I. Design*		
Approach	Method-driven with a fixed set-up; topics decided by the trainer	Need-driven with an adaptive set-up; topics decided by the participants
Content	Elementary modules: *1. Income and expenditures* Get insight into types of incomes and expenditures *2. Account book* Get insight in daily expenditures *3. Administration in order* Keeping records of financial administration *4. Making ends meet* Keeping balance between income and expenditures using budgeting *5. Get your profit* Apply for benefits and allowances *6. Solving debts* How to address arrears in payments *7. Tips for saving* How to economize on expenditures *8. Insuring, saving, and borrowing* How to decide among various financial products	Main modules:^*^ *1. Income* Types of income, additional benefits, and allowances, taxes *2. Financial administration* Banking, checking bills, dealing with a bailiff, sorting mail 3. *Making ends meet (now)* Budgeting, prioritizing, account book, resisting temptations 4. *Making ends meet (later)* Insuring, saving, borrowing

*II. Trainer*		
Role^**^	1. Transfer of information 2. Teaching skills 3. Support behavioral change	1. Support behavioral change 2. Teaching skills 3. Transfer of information
Needed competences	Clear instructions	Differentiating and activating didactics
*III. Assignments*		
	Set goal (session 1) Module-related assignments Plan for the future and stick to it (final session)	Design your program (session 1): Set goal & create an action plan Choose topics Goal-action plan Create a rule of thumb

This table summarizes the programs according to their design. Implementation might differ (see “Implementation check”). ^*^We list the main modules and some of the corresponding topics. ^**^In order of importance.

### Design of the behaviorally informed program

We designed a behaviorally informed financial education program using insights from both motivation and behavioral change theories and elements of the traditional program. Similar as for the traditional program, the purpose of the BI program was to foster healthy financial behavior. However, the intended mechanism to reach this goal differs. In designing this program, we assumed that improving financial knowledge and skills is insufficient to foster healthy financial behavior. Individuals often face ambivalence, internal and external barriers, and problems with self-regulation that may prevent behavioral change. Furthermore, the financial problems faced by financially vulnerable consumers may tax their mental capacities required for financial decision-making (Ridley et al., 2020; De Bruijn and Antonides, 2022). For these reasons, we designed a program that focuses on both improving financial skills and knowledge and supporting behavioral change.

The set-up of the BI program differs from the traditional program in three respects: (1) approach, (2) role of the trainer, and (3) assignments (see Table 1 for an overview). First, the BI program adopts a need-driven and adaptive approach, meaning that the contents of the program are adapted to the participants’ needs. This approach is apparent from the role of participants in designing the program. In the first session, the trainer presents potential topics to discuss around four modules: (1) income, (2) financial administration, (3) making ends meet (now), and (4) making ends meet (later). Thereafter, participants choose which topics they will discuss during the other sessions. The trainer designs the rest of the program accordingly. As a consequence, topics discussed differ among program rounds.

Second, we adjusted the role of the trainer. An important task of the trainer in the behaviorally informed program is to increase motivation and implementation, and to enhance behavioral change. We instructed trainers to apply elements of autonomy-supportive teaching derived from self-determination theory (Kusurkar et al., 2011). The purpose of autonomy-supportive teaching is to make participants feel autonomous, competent in their learning, and supported by their peers and trainers (enhancing relatedness), which all increase intrinsic or autonomous motivation (Ryan and Deci, 2000a,b; Kusurkar et al., 2011).^7^ We translated these insights into two elements of the program: (1) Design-your-program assignment during the first session in which participants set their own goals and choose topics for the rest of the sessions and (2) participants decide about homework by themselves. Additionally, we instructed trainers to avoid a controlling context and encourage the active participation of participants during the sessions. The role of the trainer was to create a positive, respectful, and sharing atmosphere where participants felt safe to share their stories, feelings, and questions, which supported the feeling of relatedness (Kusurkar et al., 2011).

Furthermore, trainers use the six-stage transtheoretical model of behavioral change and specific interviewing techniques as motivational instruments. The transtheoretical model proposes six stages of behavioral change: precontemplation, contemplation, preparation, action, maintenance, and termination (Prochaska et al., 1992; Prochaska and Velicer, 1997). Several studies have adapted this model to the context of financial behaviors and financial education (for an overview, see Peeters et al., 2018). During the first session, the trainer discusses the model and asks participants in which stage they place themselves and which barriers they face in changing their financial behavior. In later sessions, the model functions as a stepping-stone to refer to. Additionally, trainers apply some specific interviewing techniques, such as change talk, reflective listening, and asking open questions. Change talk is a motivational interviewing technique which helps to resolve ambivalence of participants, a common aspect of behavioral change, by highlighting the differences between the current and desired status quo (Miller and Rollnick, 2002). Trainers use specific open questions (e.g., How do you do it now? How are you going to do it? And how do you keep it up?) to enhance implementation of desired behaviors.

Third, we designed implementation assignments to enhance behavioral change. Each program session ends with making a goal-action plan and creating a rule of thumb. In a goal-action plan, participants define a session-related goal, describe the planned activities to achieve that goal, describe what and who they need for performing the activities, and set a deadline. These plans may help participants to develop concrete and actionable goals and to apply them in practice. Rules of thumb are simple heuristics of routines for financial decision-making (e.g., “I save 50 euros every month for unexpected expenditures”) and are easy to recall and to implement. Drexler et al. (2014) found that a rule-of-thumb-based financial education program for micro-entrepreneurs worked better than a standard accounting approach. Because universal rules of thumb do not address the complexity of the context for consumer financial decisions (Willis, 2008), we chose to use self-created rules of thumb as an assignment. To create enough room for these new elements, the BI program pays less attention to the transfer of knowledge, while keeping the length of the program about the same as the traditional program.

In other respects, the set-up of the BI and the traditional programs are similar. Before roll-out, we tested the BI program in a field setting and improved the program materials. Thereafter, we instructed the trainers of the BI program regarding the foundations, design, and key elements of this program during a full-day training session. Trainers taught either the BI or the traditional program, not both.

## Materials and methods

### Quasi-experimental design

**Figure 1:**
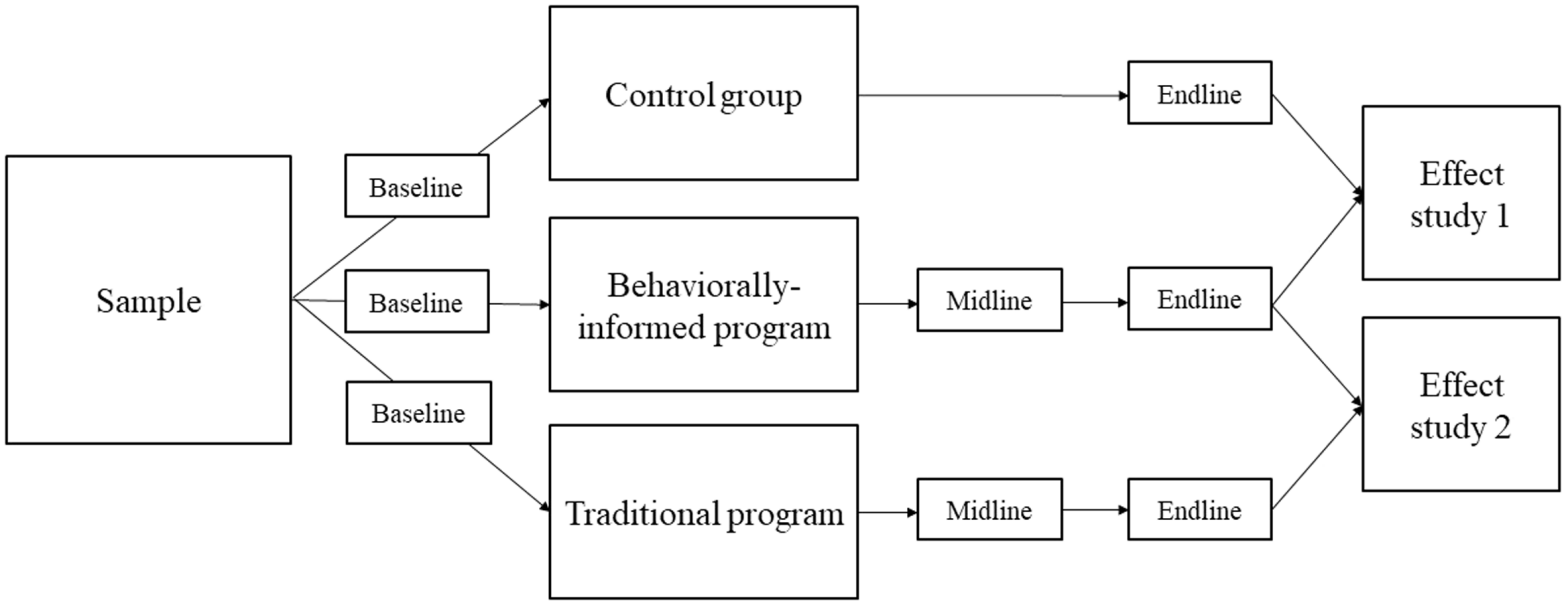
Quasi-experimental research design. Effect study 1 compared the behaviorally informed program group with the control group. Effect study 2 compared the behaviorally informed with the traditional program group. Baseline survey: before the start of the program; midline survey: during the last session; endline survey: 6 months after the program’s start.

Figure 1 shows the quasi-experimental design of our study, which consisted of three conditions: (1) a traditional financial education program group, (2) a behaviorally informed (BI) financial education program group, and (3) a control group receiving no financial education. We conducted the field study in collaboration with five local government or debt counseling organizations. These field partners covered different areas (urban vs. rural), population sizes (ranging from cities with less than 40,000 to cities with over 300,000 citizens), and type of organization (municipality, social welfare organization). None of these field partners was able to implement all three conditions. Three field partners (indicated here by numbers 1–3) implemented both the behaviorally informed program and a control group, while two field partners (indicated by numbers 4 and 5) implemented both programs but no control group. As a consequence, the quasi-experimental design consisted of two separate effect studies. Effect study 1 focused on the effects of the behaviorally informed program compared to the control group. Effect study 2 investigated whether the behaviorally informed program was more effective than the traditional program. The design of this study was approved by the Research Committee of the Wageningen School of Social Sciences. As a consequence, this study was waived from a review by the ethical committee (in line with the formal requirements at the time we conducted this study).

### Sample and treatment assignment

Participants were recruited from September 2017 to December 2018. We implemented several program rounds for each field partner during the study period and recruited participants before each program round,^8^ in close connection with the organizational processes of each field partner. As a consequence, recruitment processes differed among the field partners. Field partner 1 recruited participants during locally organized recruitment events for which they invited a broad target population (welfare claimants, debt service clients, and clients of local welfare organizations). Field partners 2, 3, and 5 mainly recruited participants among their clients (clients of budget management, debt services, and local welfare organizations) *via* their professionals. For field partner 5, some clients participated in the program to complete their budget management trajectory. For field partner 4, most participants enrolled in a financial education program as part of their debt reduction trajectory. We classified the participants as financially vulnerable, considering the recruitment process (mainly among debt service clients and welfare claimants) and the baseline sample characteristics (see “Sample information and balancing”).

All potential participants were informed about the purpose of the study, assignment procedures (if applicable), expected efforts (completing surveys), incentives for completing the survey, anonymous processing of the data, and the voluntary character of participation in the study. If people agreed to participate in the study, they signed an informed consent form. A total of 277 participants signed the consent form and completed the baseline survey, while 174 participants also completed the endline survey.

We applied different procedures in assigning participants to the experimental conditions. For effect study 1, we planned to randomly assign participants to the experimental and control conditions as much as possible. However, this was not feasible in practice, mainly because in several program rounds, our field partners recruited too few participants to split them into both a treatment and a control group. In these cases, we assigned the main part or all participants to the BI program group to ensure that the program could start with a minimum number of participants. Holding our key principle that all control group participants would be willing to participate in a financial education program, we applied three methods to create a control group. First, some participants were randomly assigned to the control group (9 participants of the final sample). Second, we asked potential participants who were willing to participate in the program but could not start directly (e.g., for practical reasons) to participate in the control group (10 participants). Both these and the randomly assigned participants were invited to participate in the program after completing the endline survey. Third, we recruited an additional group of participants beyond the standard recruitment process. Using a mailing and face-to-face recruitment, we asked people from a similar target group (debt trajectory clients, social benefit claimants, and visitors of financial consultation hours) to participate in the study. To hold our key principle for the control group, we included an additional question in the baseline survey: “Are you willing to participate in a financial education program if you were able and had the time to participate?” We included only participants who answered “yes” to this question in the control group (14 in the final sample). From the final sample, four participants participated twice in the study; first in the control group and thereafter in the BI program group. Overall, the control group consisted of 33 participants.

For effect study 2, we assigned participants to one of the two program groups using the following procedure. For field partner 4, we asked potential participants for their preferred day part (afternoon or evening) to follow the program and assigned them accordingly. To prevent selection bias, we rotated the day part of the programs (round 1: traditional program in the afternoon; BI program in the evening; round 2: vice versa). For field partner 5, we mainly randomly assigned participants to one of the two program groups. A few participants were assigned according to their preferences for a particular program time. We note that none of the field partners informed potential participants that the programs for the different day parts differed in content. If two or more members of the same household participated in the study (effect study 1 or 2), we assigned them to the same experimental condition. In “Sample information and balancing,” we compare the conditions for both effect studies on background characteristics and outcomes.

## Data and empirical strategy

### Data collection

We collected survey (baseline, midline, and endline), administrative, observational, and interview data. The baseline survey was completed before or at the start of the first program session. A small number of participants completed this survey before the second program meeting. Participants of both program groups completed the midline survey at the end of the final program meeting. This survey contained different outcome measures than the baseline and endline surveys and focused primarily on evaluating different aspects of the program (see “Measures: Evaluation of program aspects”). The endline survey was administered about 6 months after the start of the program.^9^ We took substantial effort to increase the response of the endline survey by sending reminders per email (twice), sending text messages, and calling several times in case of non-response.

The main survey mode for all survey rounds was CAWI (Computer Assisted Web Interviewing). A small number of participants completed the surveys using paper and pencil. Thereafter, research assistants digitalized and checked the data. Participants received a cookbook or a gift card of 5 euros for completing the baseline survey and a gift card of 10 euros for completing the endline survey.

Table 2 provides an overview of the survey completion rates. A total of 277 participants completed the baseline survey. The final sample consisted of 140 participants for effect study 1 (33 subjects assigned to the control group; 107 subjects to the BI program) and 141 participants for effect study 2 (107 subjects assigned to the BI program and 34 subjects to the traditional program group). Endline response rates slightly differed among these conditions ranging from 56.9% (control group) to 68.0% (traditional program group). Additionally, we collected administrative data about program attendance, reasons for absence, and household members who also participated in the study. Furthermore, we collected observational and interview data to check whether both financial education programs were implemented according to their design. Research assistants observed a substantial part of the program sessions using a standardized observation form and interviewed the trainers of both programs.

**Table 2:** Sample size and survey completion rates per condition.

	Control group	BI program	Traditional program	Total
Completed baseline	58	169	50	277
Completed baseline and midline	2	96	32	130
In %		56.8%	64.0%	
Completed baseline and endline	33	107	34	174
In %	56.9%	63.3%	68.0%	62.8%
Program followed^*^	1	96 (83)	32 (24)	160 (139)
Program not followed^**^	32	11 (24)	2 (10)	14 (35)
				
Average number of sessions attended		3.55	3.56	

Numbers are based on the assigned treatment status. ^*^Program followed: attended at least one financial education session (reported in parentheses: attended at least three sessions). ^**^Program not followed: attended zero (in parentheses: less than three) sessions.

### Measures: Outcomes and covariates

We report a large number of outcome variables relative to the sample size. As a consequence, we expect some of the variables to show significant results due to chance. To avoid overemphasis on any single significant result, we minimized the number of outcome variables using the standardized indices procedure proposed by Kling et al. (2007) and applied in several other studies (e.g., Banerjee et al., 2015; Theodos et al., 2018). For each group of outcomes, we report a summary index. We constructed these indices first by creating scores for every single outcome, using factor analysis or principal component analysis if needed. Second, we switched the signs of each outcome such that higher scores corresponded to “better” outcomes. Next, we standardized each outcome into a *z*-score, for effect studies 1 and 2 separately, by subtracting the control group (traditional program group) mean at the corresponding survey round and dividing by the corresponding control (traditional program) group standard deviation. Then, we created five groupings of outcomes, each reflecting a specific domain, and averaged the *z*-scores. Again, we standardized it against the control (traditional program) group’s mean and standard deviation. As a result, the estimators could be interpreted as effect sizes in standard deviation units relative to the control (traditional program) group. Below we briefly discuss the indices, underlying individual outcomes, and used covariates (see Supplemantary material for all items).

#### Financial skills and knowledge

We measured this variable using a one-dimensional scale (*α* = 0.909)^10^ including four applied knowledge-focused items (e.g., “I know which letter or email I should keep and which I can throw away”) and four applied skills-focused items (e.g., “I know how to make a budget”).^11^ Principal component analysis yielded a single component explaining the eight items.

#### Financial-psychological indicators

This index consisted of three parts: financial attitude, financial-psychological self-evaluation, and motivation. We measured financial attitude using two items that addressed the attitude of participants toward spending money (OECD, 2015). As this measure is commonly used in financial education program evaluations, we decided to include this measure despite its low reliability (*r* = 0.586). Financial-psychological self-evaluation included three items (*α* = 0.838) measuring financial self-efficacy (Lown, 2011), perceived control (Van Dijk et al., 2020), and difficulties with self-control in keeping track of one’s financial affairs. Lastly, we measured motivation using a single item: “I find it important to keep track of my financial affairs.”

#### Financial behavior

The index of financial behavior consisted of three different scales: budgeting, keeping track, and consuming consciously. We measured budgeting using four items developed by Kempson et al. (2013), asking whether one makes a budget, how often and accurately one does this, and whether one sticks to it.^12^ The keeping-track scale consisted of five items (*α* = 0.845) measuring to what extent the respondents keep track of their financial affairs. The consuming-consciously scale included three items (*α* = 0.844) about consciously spending money.

#### Financial well-being

This index consisted of three outcomes: chronic financial stress, general financial stress, and a financial well-being scale. Chronic financial stress was measured using a six-item scale (*α* = 0.939), reflecting different stress symptoms related to one’s financial situation. General financial stress was measured by a single item: “In the last month, how often did you experience stress due to your financial situation?” We used the abbreviated scale (*α* = 0.727) of the Consumer Financial Protection Bureau (2015, 2017) to measure financial well-being.^13^ According to their definition, financial well-being refers to a state in which one can fully meet current and ongoing financial obligations, feels secure, and is able to make choices to enjoy life.

#### Financial situation

This index included three aspects of one’s household financial situation. We measured relative financial buffer using an item about how long one’s household can still pay the groceries and fixed charges when the main income source would be lost (derived from OECD, 2015). Additionally, we measured how well one’s household was able to make ends meet (Eurostat, 2014) and perceived debts by asking whether one thought one’s household had excessive debts (Lusardi and Mitchell, 2018).

#### Covariates

Additionally, we collected survey data on age (in years), gender (male), educational level (lower, intermediate, and higher educated), migration background (dummy variable), partner, children (dummy variable), net household income (log),^14^ and main household income source (dummy variable: income from employment or self-employment versus other types of income). Additionally, we included a dummy variable for receiving either professional assistance for financial management (assistance from an administrator or budget holder) and/or professional debt assistance (debt rescheduling scheme or amicable debt settlement). Finally, items about income, living together with a partner, and with children, were included both in the baseline and in the endline survey.

### Measures: Evaluation of program aspects

The midline survey provides insight into the differences between both programs as perceived by the participants, with respect to three categories of outcomes.

#### Trainers’ teaching behavior

Building upon the validated teaching behavior scale of Maulana et al. (2015), measures for each of two domains of trainers’ teaching behavior were constructed: (1) clear instruction and creating a safe learning environment (*α* = 0.932) and (2) adaptive teaching and activating learning (*α* = 0.909).^15^ We expected similar scores between both programs on the first dimension and higher scores for the BI program on the second dimension.

#### Program evaluation

We measured two aspects of participant’s evaluation of the program: (1) perceived usefulness of (elements of) the program (*α* = 0.821) and (2) overall satisfaction with the program (single item). See Supplemantary material for the measurement instruments.

#### Perceived improvement in outcomes

Participants were asked whether the program contributed to (1) improving financial management (four items, *α* = 0.887), (2) improving financial-psychological indicators (four items, *α* = 0.864), and (3) providing and implementing implementation tools (two items: rules of thumb and goal-action plans, *α* = 0.722) designed for the BI program. We expected similar scores on the first and higher scores for the BI program on the second and third aspects. See Supplemantary material for the measurement instruments.

### Sample information and balancing

Table 3 shows the descriptive statistics of our full sample and for each condition separately. Nearly all participants in our study were financially vulnerable. The average net household income was 1,424 euros per month, which is around the Dutch monthly minimum wage. Most participants (64%) relied on social benefits (social welfare, disability, unemployment, or pension) as the main income source. Only one-third of our sample (36%) earned income from (self-)employment. Furthermore, participants were mainly lower (47.0%) or intermediate (40%) educated. Only a small part was higher educated. More than two-thirds of the sample received professional assistance for financial management and/or problematic debts. Most participants were female (58%); the average age was 43.3 years. Only a small part of our sample had a partner (22%), while about one-third had one or more children living at home (36%).

**Table 3:** Sample descriptive statistics and balancing.

		Descriptive statistics			Balancing	
	Full sample	Control	BI program	Traditional program	BI program vs. control	BI vs. trad. Program
	(1)	(2)	(3)	(4)	(5)	(6)
Male	0.42	0.42	0.39	0.5	−0.03	−0.09
	(0.49)	(0.5)	(0.49)	(0.51)	(0.1)	(0.1)
					[0.75]	[0.36]
Age	43.32	41.03	43.46	45.11	2.43	−0.65
	(12.38)	(14.17)	(12.12)	(11.3)	(2.71)	(2.3)
					[0.37]	[0.78]
Migration background	0.26	0.3	0.27	0.21	−0.03	0.07
	(0.44)	(0.47)	(0.45)	(0.41)	(0.09)	(0.09)
					[0.73]	[0.40]
Lower education	0.47	0.52	0.45	0.5	−0.07	−0.03
	(0.5)	(0.51)	(0.5)	(0.51)	(0.1)	(0.1)
					[0.51]	[0.76]
Intermediate education	0.4	0.21	0.44	0.47	0.23	−0.05
	(0.49)	(0.42)	(0.5)	(0.51)	(0.09)	(0.1)
					[0.01]	[0.61]
Higher education	0.12	0.24	0.11	0.03	−0.13	0.08
	(0.33)	(0.44)	(0.32)	(0.17)	(0.08)	(0.05)
					[0.11]	[0.07]
Income from (self-)employment	0.36	0.3	0.42	0.24	0.12	0.16
	(0.48)	(0.47)	(0.5)	(0.43)	(0.09)	(0.09)
					[0.20]	[0.08]
Partner	0.22	0.12	0.25	0.24	0.13	−0.01
	(0.42)	(0.33)	(0.44)	(0.43)	(0.07)	(0.09)
					[0.07]	[0.91]
Children	0.36	0.33	0.38	0.32	0.05	0.04
	(0.48)	(0.48)	(0.49)	(0.47)	(0.1)	(0.1)
					[0.60]	[0.67]
Professional assistance	0.69	0.52	0.7	0.82	0.18	−0.16
	(0.46)	(0.51)	(0.46)	(0.39)	(0.1)	(0.08)
					[0.07]	[0.05]
Household income (in euro)	1,424.23	1,312.07	1,504.25	1,284.56	192.18	202.55
	(566.91)	(500.03)	(632.78)	(337.38)	(111.72)	(89.78)
					[0.09]	[0.03]
						
Joint test (*p*-value)					0.31	0.07
						
*N*	163–174	29–33	100–107	34	129–140	121–128

We report here the descriptive statistics for the sample that completed both the baseline and endline survey. Descriptive statistics for the sample after baseline survey completion were quite similar. Columns (1)–(4) show covariate means with standard deviations in parentheses for the full sample and the experimental groups separately. Columns (5) and (6) show the outcome of regressing each background characteristic on a dummy indicating treatment assignment. The coefficient provides the difference scores between the BI program and control (5) or traditional program (6) group. Standard errors are shown in parentheses; value of ps in brackets. The second last row shows value of ps of a joint hypothesis test.

Additionally, we tested whether the groups of both effect studies were balanced. For effect study 1, the BI program group seemed to be well balanced compared to the control group for several background characteristics, but less well for education level, professional assistance, household income, and partner. For the BI program compared to the traditional program group (effect study 2), differences in baseline characteristics were more pronounced, specifically for income, professional assistance, and income from (self-)employment. To control for these imbalances, we decided to include the above-listed variables as covariates in our regressions. Additionally, we performed a robustness check in which we matched subjects on all background characteristics (see “Empirical methods”).

### Empirical methods

We observed non-compliance for effect study 1 (see Table 2) and non-take-up among both financial education programs in effect study 2. To adequately incorporate non-compliance and non-take-up in our empirical strategy, we estimated three types of treatment effects: (1) intention-to-treat effects (effect study 1 and 2), (2) local average treatment effects (effect study 1), and (3) treated-only effects (effect study 2).

To estimate intention-to-treat (ITT) effects, we used a difference-in-difference (DID) approach. For the ITT analysis, we compared participants assigned to the BI program group to those assigned to the control group (effect study 1) or the traditional program group (effect study 2), irrespective of whether they received the treatment or not.^16^ The ITT-model estimates the effects of offering the BI program and takes the following form:


(1) $$Y_{it}=\beta_{0}+\beta_{1}Z_{i}+\beta_{2}A_{t}+\beta_{3}Z_{i}\times A_{t}+X_{it}\delta +\epsilon_{it}$$

$Y_{it}$ is the outcome variable for individual i in period t. $Z_{i}$ is a binary variable indicating the assigned treatment status for each unit (0 = control or traditional program group, 1 = BI program group). $A_{t}$ is a time dummy variable (0 = baseline, 1 = endline). The parameter of interest is $\beta_{3}$ which indicates the DID estimator; $\epsilon_{it}$ is the error term. The vector $X_{i}$ contains the covariates. To ensure enough observations per estimated coefficient, we reduced the number of covariates per specification to a maximum of three. Following the balance performance (“Sample information and balancing”), we included household income (log), professional assistance, and education level as covariates in the main specification, and income from (self-)employment, partner, and migration background in a robustness check. If available, we included both baseline and endline values for the covariates. According to Lechner (2010), time-varying covariates (if not affected by the treatment) perform better in removing time-confounding than only including pre-treatment measures. Following the recommendations of Abadie et al. (2017), we clustered standard errors on the household level as we assigned participants who belonged to the same household to the same treatment group. For the analyses of the DID treatment effects, we used the diff-package designed by Villa (2016).

For effect study 1, we additionally estimated local average treatment effects (LATE) in a two-stage least squares (2SLS) framework which adequately addresses potential selection effects caused by non-compliance. This model assumes that all effects operate *via* treated individuals (i.e., subjects that participated in the financial education program). Following the guidelines of Gerber and Green (2012), we counted partially treated subjects as fully treated. In the first stage, we used the assigned treatment status ($Z_{i}$) as an instrumental variable for the actual treatment status ($T_{i}$). The first stage equation takes the following form:


(2) $$\begin{array}{c} \hat{T_{i}}=\alpha +\mu Z_{i}+X_{i}\delta +\epsilon_{i} \end{array}$$

In the second stage, we used the following equation to estimate the treatment effects:


(3) $$\begin{array}{c} Y_{i}=\beta_{0}+\beta_{1}\hat{T_{i}}+X_{i}\delta +\epsilon_{i} \end{array}$$

The parameter of interest is $\beta_{1}$ which indicates the treatment effect among compliers. Compliers are subjects who only participated in the treatment (BI program) if they are assigned to this treatment. The vector $X_{i}$ contains the same covariates as for the difference-in-difference analysis (see Equation 1) and additionally included the baseline scores for the outcome of interest.

For effect study 2, the 2SLS-apprach was not feasible due to non-take-up among both financial education program groups. As an alternative solution, we estimated a treated-only model in which we compared educated participants of the BI program with educated participants of the traditional program.^17^ The treated-only model estimates the effects of participation in the behaviorally informed program and provides insight into the efficacy of the BI program, which is relevant for policymakers and practitioners. We used the following equation:


(4) $$\begin{array}{c} Y_{it}=\beta_{0}+\beta_{1}T_{i}+\beta_{2}A_{t}+\beta_{3}T_{i}\times A_{t}+X_{it}\delta +\epsilon_{it} \end{array}$$

Equation (4) is very similar to equation (1). The difference is that we used the *actual* treatment status ($T_{i}$) for each unit (0 = traditional program group, 1 = BI program group) rather than the *assigned* treatment group ($Z_{i}$). We defined educated participants as those who completed at least one (main analyses), respectively three (robustness check) program session(s).

To verify whether choices in model specification and estimation did affect our results, we performed several robustness analyses in which we varied (1) added covariates and (2) including/excluding the participants of particular field partners. Additionally, we performed a propensity score matching difference-in-difference model to measure ITT and treated-only effects. In estimating the propensity score, we used a rich set of control variables (see Table 3) that might affect both treatment assignment, program participation, and (any of) the outcomes.

The statistical power of our study may have been reduced by three factors. First, our final sample was relatively small, despite intensive recruitment and survey response campaigns. Second, among those who were assigned to either the traditional or BI program group, some did not complete any of the financial education sessions (see Table 2). Furthermore, one participant of the control group attended the financial education sessions. Third, participants of both programs attended less (on average 3.6 sessions) than the planned five-to-six sessions. Due to these challenges, we expected difficulties with detecting potential treatment effects for outcomes possibly resulting after a change in financial behavior (financial well-being and financial situation) and for detecting significant differences between the BI and traditional programs. In attempting to solve these problems, we decided to minimize the number of outcomes using standardized indices (see “Measures: Outcomes and covariates”). Furthermore, we decided to apply different empirical methods and specifications to avoid overreliance on any single method or specification (as discussed above). We interpret effects only as such if they were robust, which is significant under different specifications.

To estimate the effects on evaluation scores of program elements (using the midline survey; see “Measures: Evaluation of program aspects”), we applied propensity score matching using a set of control variables measured in the baseline survey. We performed these estimations using psmatch2 (Leuven and Sianesi, 2003) without clustering the standard errors.

## Results

### Implementation check

We used observational and interview data to evaluate the implementation of both financial education programs. The traditional program was largely implemented according to its design. The trainers of this program discussed all main topics of the workbook more or less in the same order. Sometimes, they discussed additional modules provided in the course materials like “varying income,” “money and children,” and “money and relationships.” As part of their homework, the trainer asked the participants to keep track of their earnings and expenditures during the program period. We found differences across traditional program trainers in teaching the set-a-goal assignment (not taught vs. more prominent role) and seeking interaction with participants (more vs. less prominent role). The trainers involved in our study were experienced and had already run this program for several years.

Most elements of the BI program were implemented according to its design. Indeed, trainers paid much attention to the “design-your-program” assignment. The participants’ main chosen topics were budgeting, book accounting, financial administration, and resisting temptations. Furthermore, we found that trainers indeed discussed the stages-of-change model, applied elements of the (motivational) interviewing techniques, and discussed the goal-action-plan assignment. Some deviations from the program design were observed. First, during the “design-your-program” assignment, some trainers steered participants to choose topics they found relevant (e.g., budgeting). Second, most trainers did not deliver the rules-of-thumb assignment, possibly due to time constraints. Additionally, both programs regularly had fewer than eight participants. Overall, these deviations from the planned implementation in both programs might have attenuated the differences between both programs, thus limiting the experimental manipulation for effect study 2.

### Results effect study 1: Treatment effects of the behaviorally informed program

**Figure 2:**
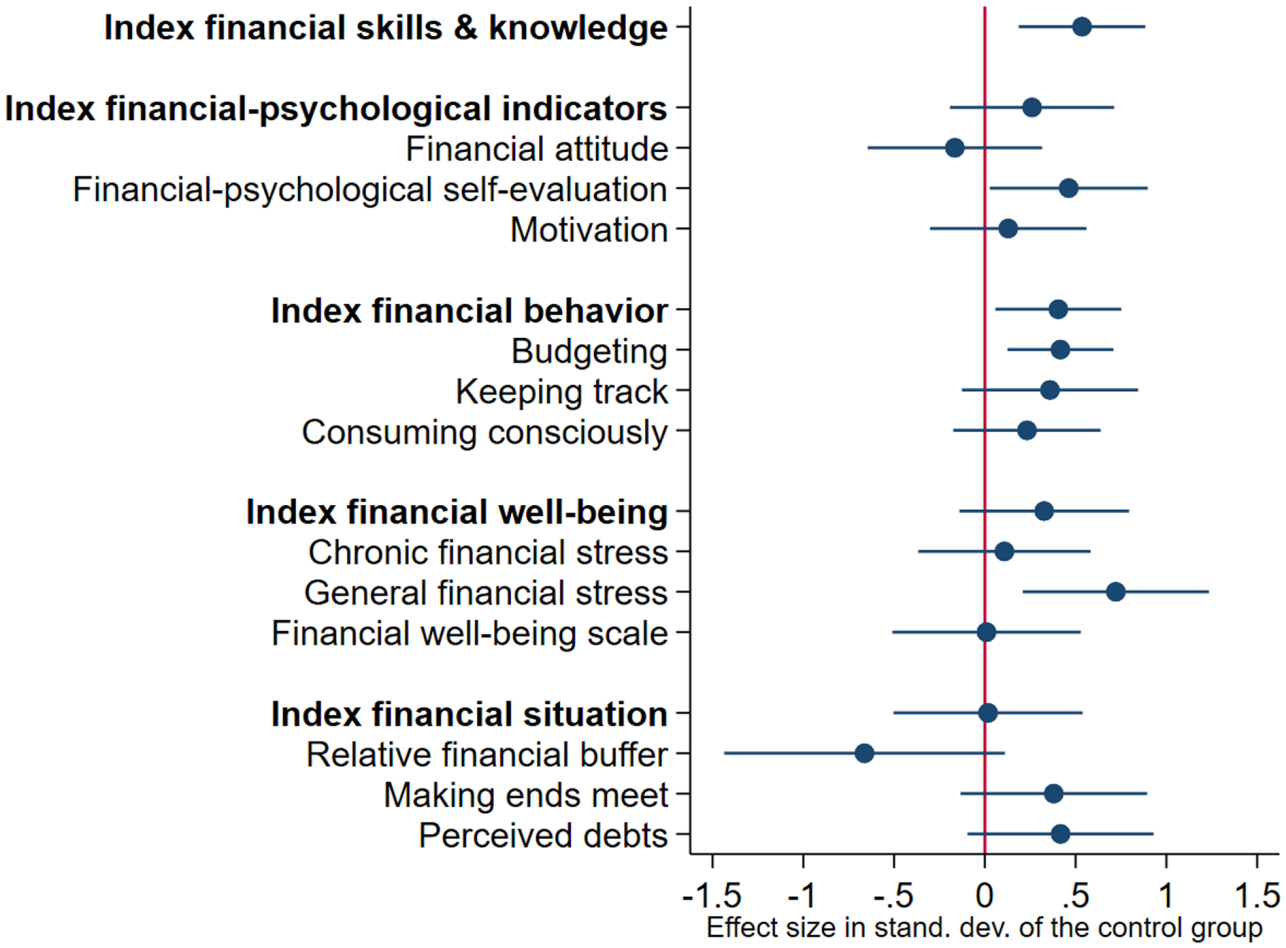
Behaviorally informed program versus control group: Intention-to-treat effects. This figure summarizes the intention-to-treat effects for the five primary outcomes (see Table 4, column 1). All treatment effects are presented as standardized *z*-scores, standardized to the control group. Each entry shows the standardized outcome and its 95% confidence interval.

Figure 2 and Table 4 provide an overview of the ITT effects of the BI program in comparison with the control group. We found significant effects of being assigned to the BI program on financial skills and knowledge (ITT = 0.536, SE = 0.176, *p* = 0.003) and financial behavior (ITT = 0.405, SE = 0.175, *p* = 0.023). Thus, *offering* this program improved the financial skills and knowledge with 0.54 SDs and financial behavior with 0.41 SDs. These results hold under all specifications. In contrast, we did not find significant positive ITT effects on psychological outcomes, subjective financial well-being, and financial situation.

**Table 4:** Behaviorally informed program versus control group: Intention-to-treat and local average treatment effects.

	ITT		LATE	
Indexed outcomes	(1)	(2)	(3)	(4)
Financial skills & knowledge	0.536	0.467	0.761	0.620
	(0.176)	(0.170)	(0.184)	(0.177)
	[0.003]	[0.007]	[0.000]	[0.000]
Financial-psychological outcomes	0.260	0.137	0.535	0.439
	(0.228)	(0.212)	(0.234)	(0.212)
	[0.257]	[0.519]	[0.022]	[0.039]
Financial behavior	0.405	0.452	0.558	0.509
	(0.175)	(0.177)	(0.183)	(0.181)
	[0.023]	[0.012]	[0.002]	[0.005]
Subjective financial well-being	0.327	0.294	0.348	0.388
	(0.236)	(0.213)	(0.221)	(0.200)
	[0.169]	[0.171]	[0.115]	[0.053]
Financial situation	0.018	0.031	0.055	−0.039
	(0.263)	(0.237)	(0.221)	(0.201)
	[0.947]	[0.896]	[0.802]	[0.847]
				
Difference-in-difference	Yes	Yes	No	No
IV-2SLS	No	No	Yes	Yes
				
Observations	244–250	266–272	244–250	266–272

Column (1) and (2) represent mean standardized intention-to-treat effects with cluster-robust standard errors in parentheses and corresponding *p*-values in brackets. Column (3) and (4) represent mean standardized local average treatment effects with clustered standard errors in parentheses and corresponding p-values in brackets. Specifications (1) and (3) include log income, education level, and professional assistance as covariates. Specifications (2) and (4) include partner, income source, and migration background as covariates. Specifications (3) and (4) include also the baseline score of the outcome variable as covariate. Standard errors are clustered at the household level. Differences in number of observations within a specification are caused by missing values.

**Figure 3:**
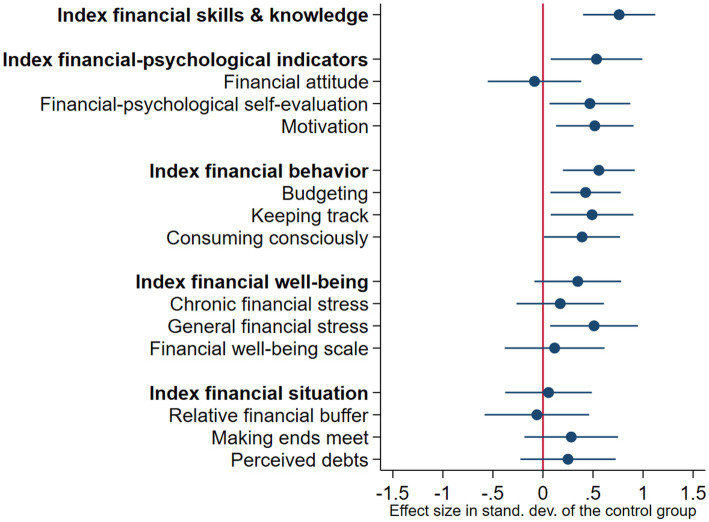
Behaviorally informed program versus control group: Local average treatment effects *Notes*. This figure summarizes the local average treatment effects for the five primary outcomes (see Table 4, column 3). All treatment effects are presented as standardized *z*-scores, standardized to the control group. Each entry shows the standardized outcome and its 95% confidence interval. The effects on financial-psychological self-evaluation, keeping track and general financial stress were not robust under alternative specifications.

Figure 3 and Table 4 show the estimates of the LATE effects of the BI program. We found robust significant positive effects on financial behavior (LATE = 0.761, SE = 0.184, *p* < 0.001), financial-psychological outcomes (LATE = 0.535, SE = 0.234, *p* = 0.022), and financial behavior (LATE = 0.558, SE = 0.183, *p* = 0.002). Thus, the effect on financial-psychological outcomes was significant under the LATE-model, but not under the ITT-model. In line with our expectations, the size of the LATE effects was larger than the ITT effects.

We conducted the following analyses to check the robustness of our ITT and LATE results: (1) applying a propensity score matching difference-in-difference method, (2) dropping participants of field partners that did not implement a control group, (3) dropping household income as covariate (to avoid missing values), (4) including two dummy variables for professional assistance (receiving financial management assistance and receiving debt assistance), and (5) dropping participants who participated both in the control and BI program group. Our results were robust under these alternative specifications.

We conducted exploratory analyses to investigate the ITT and LATE effects on all single outcomes. We found that positive effects on budgeting (ITT = 0.416, SE = 0.147, *p* = 0.006; LATE = 0.426, SE = 0.179, *p* = 0.017) mainly have driven the effect on financial behavior. The effects on the other aspects of financial behavior (keeping track and consuming consciously) were also positive, but not significant, as reflected in Figure 2. Additionally, we found that positive effects on motivation (LATE = 0.518, SE = 0.198, *p* = 0.009) were the main driver of the LATE effect on financial-psychological outcomes. We did not find robust significant ITT or LATE effects on other individual outcomes.

### Results effect study 2: Effects behaviorally informed versus traditional program

**Figure 4:**
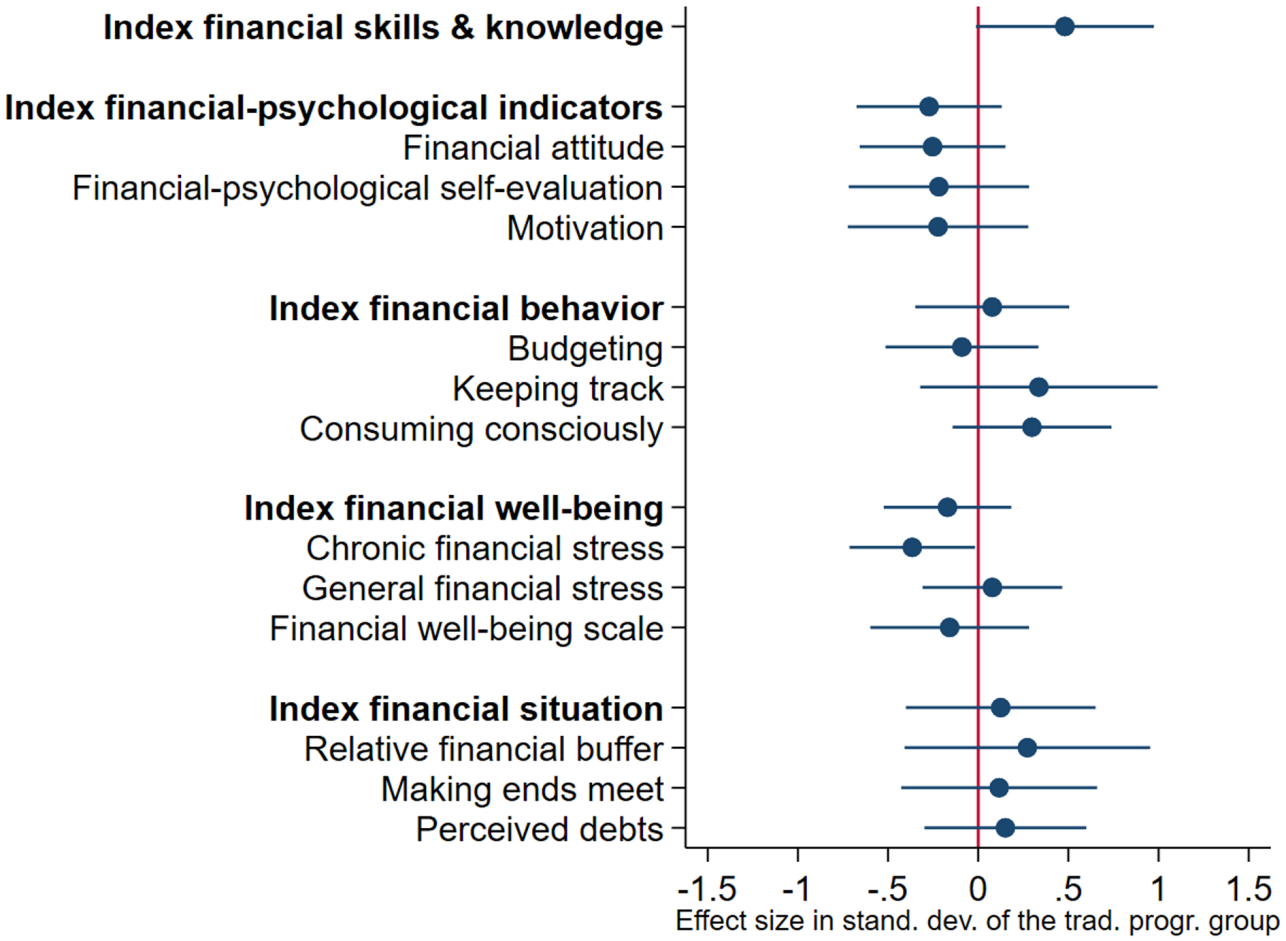
Behaviorally informed (BI) versus traditional program: Treated-only effects. This figure summarizes the treated-only effects for the five primary outcomes (see Table 5, column 1). Treatment effects are presented as standardized *z*-scores, standardized to the traditional program group, and its 95% confidence interval.

Figure 4 and Table 5 display the treated-only effects of the BI program in comparison with the traditional program. We did not find evidence for effects of *participating in* the BI program compared to the traditional program for any of the outcomes. None of the outcomes were significant under more than one specification. The non-results were robust under (1) alternative specifications (same as for effect study 1), (2) the ITT-model, and (3) treatment assignment based on three or more sessions attended. We exploratively analyzed the effects on all 13 individual outcomes. We did not find robust effects on any of these outcomes.

**Table 5:** Behaviorally informed versus traditional program: Treated-only and intention-to-treat effects.

	Treated-only		ITT	
Indexed outcomes	(1)	(2)	(3)	(4)
Financial skills & knowledge	0.481	0.490	0.452	0.463
	(0.249)	(0.255)	(0.230)	(0.235)
	[0.056]	[0.057]	[0.051]	[0.051]
Financial-psychological indicators	−0.272	−0.324	−0.346	−0.394
	(0.203)	(0.198)	(0.194)	(0.189)
	[0.183]	[0.104]	[0.076]	[0.039]
Financial behavior	0.078	0.058	0.071	0.066
	(0.215)	(0.216)	(0.209)	(0.208)
	[0.718]	[0.788]	[0.735]	[0.754]
Subjective financial well-being	−0.170	−0.254	−0.276	−0.351
	(0.178)	(0.178)	(0.175)	(0.174)
	[0.342]	[0.156]	[0.117]	[0.046]
Financial situation	0.125	0.049	0.074	0.003
	(0.265)	(0.257)	(0.241)	(0.236)
	[0.639]	[0.849]	[0.761]	[0.991]
				
Observations	228–232	242–246	234–238	268–272

Column (1) and (2) represent mean standardized treated-only effects of the BI program in comparison with the traditional program. Column (3) and (4) represent mean standardized intention-to-treat effects of being assigned to the BI program in comparison with the traditional program. Cluster-robust standard errors are presented in parentheses and corresponding p-values in brackets. Column (1) and (3) include income, education level, and professional assistance as covariates. Column (2) and (4) include partner, income source, and migration background as covariates. Standard errors are clustered at the household level. Differences in number of observations within a specification are caused by missing values.

### Additional analyses

We conducted additional analyses to find out why we did not find differences between the BI and the traditional program. First, we explored the treated-only effects of participating in the BI program on evaluation scores of program elements (using the midline survey) compared to participating in the traditional program (see Table 6). We expected that participants of this program would score relatively better on financial-psychological outcomes, adaptive teaching and activating learning (evaluation trainer), and use of implementation tools than participants of the traditional program. However, neither the effects on these outcomes nor the effects on the other outcomes were significant. We note that the participants of the traditional program provided relatively high mean scores on the outcomes, suggesting little room for higher scores in the BI program. These results suggest that, in terms of outcomes for the participants, the BI and the traditional program performed more or less equally.

**Table 6:** Behaviorally informed versus traditional program: Treated-only effects on evaluation scores of program elements.

	Traditional program mean (SD)	(1)	(2)
*Trainers’ teaching behavior*			
Clear instruction & safe learning climate	4.63	−0.042	−0.049
	(0.46)	(0.117)	(0.116)
		[0.719]	[0.676]
Adaptive teaching & activating learning	4.31	0.050	0.042
	(0.47)	(0.119)	(0.115)
		[0.672]	[0.713]
*Program evaluation*			
Usefulness of (aspects of) program	4.15	0.056	0.071
	(0.69)	(0.136)	(0.149)
		[0.682]	[0.634]
Program satisfaction	3.97	0.077	0.042
	(1.43)	(0.306)	(0.308)
		[0.801]	[0.891]
*Perceived improvements in outcomes*			
Financial management	4.06	0.076	0.072
	(0.78)	(0.162)	(0.165)
		[0.642]	[0.662]
Financial-psychological indicators	4.06	0.012	0.007
	(0.79)	(0.166)	(0.172)
		[0.942]	[0.970]
Implementation tools	4.06	0.115	0.103
	(0.67)	(0.154)	(0.149)
		[0.455]	[0.490]
			
Observations		119	119

Treated-only effects of the behaviorally informed program in comparison with the traditional program with standard errors in parentheses and corresponding p-values in brackets. (1) Epanechnikov kernel propensity score matching on covariates (gender, age, education level, migration background, income source, household income, partner, children, and professional assistance), (2) same as (1) but with Gaussian as kernel matching method. Participants rated all outcomes on a scale of 1s–5.

Second, we investigated the role of teaching experience as a potential determinant of evaluation scores on program elements. The rationale is that the BI program trainers did not have experience in teaching this program, while the traditional program trainers did have that experience. A lack of BI teaching experience might have negatively affected the BI program scores explaining the non-results for the comparison between both programs. To examine this potential explanation, we considered two types of teaching experience that might have played a role. First, we considered ex-ante teaching experience. We analyzed whether BI program trainers with previous experience in teaching financial education did obtain higher scores than teachers without this experience. Second, we considered on-the-road teaching experience. Trainers taught multiple program rounds and consequently became more experienced in teaching the BI program. We investigated whether BI program trainers obtained higher scores in later program rounds. Overall, we found that neither ex-ante teaching experience nor on-the-road experience in teaching the behaviorally informed program significantly affected participants’ evaluation scores on the program elements.

## Discussion

This study aimed at designing a behaviorally informed financial education program targeting financially vulnerable consumers and investigating its impact compared with both a control group and a traditional financial education program. Below, we discuss the main findings, implications, and limitations of our study.

Our study shows three main findings. First, the behaviorally informed program had a positive effect on financial behavior as compared with the control group. We found that *offering* the BI program improved financial behavior with 0.41 SDs (ITT effect) while the improvement for compliers was 0.56 SDs on average (LATE). Additionally, we found a significant treatment effect of 0.54 SDs (ITT), respectively, 0.76 SDs (LATE) on financial skills and knowledge. For financial-psychological outcomes, the treatment effect was only significant under the LATE-model (0.54 SDs). These results are held under several robustness specifications. Explorative analysis suggested that the effect on financial behavior was mainly explained by a positive effect on budgeting. In terms of program intensity, target population, and study design, our study is most comparable to Collins (2013). This study found positive effects of a mandatory financial education program on some self-reported behaviors, specifically paying bills on time and planning for the future, but no consistent effects on budgeting. A potential explanation of why we found positive effects on budgeting specifically is that budgeting was among the common activities of the BI program. Furthermore, the motivational and implementation components of the program might have contributed to start, improve, and sustain this activity. Financial skills, knowledge, financial-psychological outcomes, and behaviors may be considered direct effects of the BI program because these elements were taught during the program sessions.

Second, we did not find evidence for positive effects on financial well-being and financial situation. A potential reason is that our sample size was too small to detect effect sizes that can still be meaningful. Given our sample size, *α* = 0.05, β = 0.2, and the estimated standard errors the *post-hoc* minimum detectable effect sizes (MDE) for these outcomes were.66 and.74 SDs, respectively. Furthermore, we should expect smaller effect sizes for the latter two outcomes because these are indirectly related to the financial education program and may require more time and education to develop. We note that financial behavior is positively associated with both financial well-being (*r* = 0.286, *p* < 0.001) and financial situation (*r* = 0.274, *p* < 0.001). This is in line with the literature that predict and find a positive impact of financial behavior (e.g., budgeting) on financial well-being and financial outcomes (e.g., Zhang et al., 2021). For example, improved budgeting practices might help to take control over one’s finances, avoid overspending, and save for long-term goals (Zhang et al., 2021). As a consequence, one’s financial well-being and financial situation might improve. In line with this reasoning, the BI program improved budgeting and might have positive effects on these outcomes in the longer run. However, our study is not able to provide a final answer. Future studies should address this issue.

Third, our study did not find evidence that the BI program performed better than the traditional program on the primary outcomes. Given our sample size, we were able to detect effect sizes between 0.50 SDs and 0.74 SDs. As smaller effect sizes can still be meaningful, our results cannot provide a final answer whether the BI program is more effective than the traditional program. However, we even did not find significant differences between both programs for the program evaluation measures. These findings suggest that the perceived differences between both programs were smaller than expected. A potential explanation is that both programs contained significant overlap in topics discussed and education time. Additionally, both low attendance rates and deviations in implementation relative to program designs might have attenuated the (experienced) differences between both programs. Furthermore, the traditional program already obtained high scores from participants, thus leaving little room for improvement. As a consequence, we cannot provide a final answer as to whether the new elements contributed to the effectiveness of the BI program.

We discuss some shortcomings of our study. First, the evaluation period of 6 months between treatment and measuring outcomes is short. We recommend future studies to implement a longer evaluation period, preferably of at least 18–24 months, to pick up possible indirect effects on financial situation and financial well-being. Second, we report a low reliability for the commonly used financial attitude measure. Third, we faced difficulties with the implementation of the field study. We were not able to fully randomize the allocation of participants to the treatment groups. As a consequence, we cannot rule out all potential threats affecting selection, despite our efforts to increase the credibility of the common trend assumption. Additionally, we may have faced a lack of statistical power due to difficulties with recruiting participants, attrition, and partial compliance. An ideal solution to solve these problems is to run an RCT including a substantially larger sample. However, both our study and Collins (2013) show that this is not that simple. Due to the hard-to-reach target population and complex context (different governmental interventions at the same time), it will be hard to have full control over all potential threats affecting the design, implementation, and results of the study. A natural experiment might be a better solution, although this might come with selection problems and difficulties in collecting survey data. To instigate future research to the BI program in different settings, we make available the elementary course materials of this program.^18^

We end with an important puzzle. Low take-up and high drop-out are essential problems for financial education programs targeting financially vulnerable consumers (Collins, 2013; Kaiser and Menkhoff, 2017; Theodos et al., 2018). We faced the same problems. Despite considerable efforts (e.g., advertisements, mailings, and recruitment *via* professionals) of our field partners in reaching the target population, we faced low take-up and considerable drop-out rates. These issues may reflect a low demand for financial education. Furthermore, not participating can be rational as financial education does not benefit everyone (Lusardi et al., 2020a). These problems affect the cost-effectiveness of financial education programs. An effective program might not be cost-effective if too few people participate. A remaining issue is how to reach this target population. Consequently, policymakers and practitioners may consider alternative strategies to foster healthy financial behavior and to improve financial well-being. For example, they may encourage the use of budgeting tools building on commitment strategies and mental accounting (Dolan et al., 2012), as budgeting seems to improve financial well-being (Zhang et al., 2021).

In their meta-study, Kaiser and Menkhoff (2017) raise two remaining problems. First, how can we improve the effectiveness of financial education programs? Second, how can we effectively reach people who do not participate? These problems are especially pressing for reaching financially vulnerable consumers. Our work addresses the first issue and suggests that a modest financial education intervention incorporating behavioral insights has a modest positive impact on the financial skills and knowledge and the financial behavior of this target population. This result is hopeful as meta-studies have found only (very) small effects of financial education interventions on financial behavior of financially vulnerable people (Fernandes et al., 2014; Kaiser and Menkhoff, 2017).

## Data availability statement

The datasets presented in this study can be found in online repositories. The names of the repository/repositories and accession number(s) can be found below: Codes and data are available (upon request) *via* Data Archiving and Networked Services (DANS): https://doi.org/10.17026/dans-27y-s77z.

## Ethics statement

Ethical review and approval was not required for the study on human participants in accordance with the local legislation and institutional requirements. The participants provided their written informed consent to participate in this study.

## Author contributions

TM designed the behaviorally informed financial education program. E-JB and TM collected the data. E-JB performed the analyses and wrote the manuscript, while GA and TM provided feedback. E-JB, GA, and TM contributed to the design of the study. All authors contributed to the article and approved the submitted version.

## Funding

This work was supported by ZonMw (535.002.001) and by the Netherlands Organization for Scientific Research (023.005.060 to E-JB).

## Conflict of interest

The authors declare that the research was conducted in the absence of any commercial or financial relationships that could be construed as a potential conflict of interest.

## Publisher’s note

All claims expressed in this article are solely those of the authors and do not necessarily represent those of their affiliated organizations, or those of the publisher, the editors and the reviewers. Any product that may be evaluated in this article, or claim that may be made by its manufacturer, is not guaranteed or endorsed by the publisher.

## Footnotes

^1^Kaiser and Menkhoff (2017) found an effect size of 0.09 (standardized mean difference) on financial behavior and of 0.21 on budgeting and planning behavior.

^2^A recent meta-study did not find differences between low- and higher-income subjects (Kaiser et al., 2022).

^3^Some studies found that other types of financial education interventions, like financial counselling, financial coaching, and education in a matched savings account program, can improve financial outcomes for low-income participants (e.g., Agarwal et al., 2010; Grinstein-Weiss et al., 2015; Theodos et al., 2018).

^4^The description of the traditional program applies to the program provided by Nibud until 2019. Recently, Nibud has changed the set-up of the program and, on basis of the results of this study, already implemented some elements of the behaviorally informed program. See https://www.nibud.nl/kennis-in-de-praktijk/cursussen-workshops-coaching/budgetcursus-omgaan-met-geld/ for more information about course materials (in Dutch).

^5^We note that one of our field partners used their own materials based on the program developed by Nibud (before the 2019 change). This program included a fixed set-up and similar topics as the traditional program.

^6^Field partners 4 and 5 implemented, respectively, five and six traditional program sessions. We note that the average number of program sessions attended was the same for the BI and traditional program (see Table 2).

^7^We note that most people do not find elementary financial activities (e.g., budgeting or deciding about financial products) inherently interesting or enjoyable. Because intrinsic motivation for these financial behaviors is not evident, facilitating and promoting internalization is essential to enhance learning and behavioral change (Niemiec and Ryan, 2009).

^8^A program round consisted of 5–6 weeks in which the field partners either offered both programs (field partners 4 and 5) or only the BI program (field partners 1–3). In total, field partners implemented 5–9 program rounds. Overall, the field partners provided the BI program 28 times and the traditional program 12 times.

^9^A longer evaluation period was not feasible within the restricted time frame of the funding by the Dutch science organization ZonMw.

^10^Reported Cronbach’s Alphas (a) and Spearman-Brown coefficient (*r*) were estimated using all baseline surveys.

^11^The literature lacks a validated test that measures applied financial skills and knowledge in the context of our study. As a consequence, we decided to include these self-reported measures.

^12^The budgeting score was computed by the procedure proposed by Kempson et al. (2013). Because the individual items are artificially coherent (one item ends up in all subscales), Cronbach’s alpha is inflated.

^13^The original scale consisted of five items. We excluded one item ("I am just getting by financially") because it could be interpreted ambiguously.

^14^Income was measured in six categories. We used the mean of each category (upper limit of lowest category, lower limit of highest category) as income level. We estimated the logarithm of this level and used this number in our analyses. To avoid missing values for income at the endline, we imputed baseline income values for ten participants. None of these participants reported changes in partner status, which underpins the reliability of the imputation.

^15^We found these two components after including all nine items in a principal component analysis.

^16^Due to survey attrition, not all initially assigned participants were included, thus slightly modifying the ITT-design. Survey attrition did not seem to be systematic, as descriptive statistics after baseline survey completion were quite similar to that after baseline and endline survey completion, both overall and per condition.

^17^We determined non-compliance using the attendance registration of the field partners. As an additional check, we asked in the endline survey whether respondents participated in a financial education program in the last six months. Four non-compliers of the BI program group (ITT) reported to have participated in a program, while they were non-compliers according to the administrative data. To ensure that the control group only contained non-educated participants, we dropped these participants from the treated-only analysis for effect study 1.

^18^Elementary course materials of the BI program are available (in Dutch) upon request from the corresponding author.
