# Supplementary material for: A behaviorally informed financial education program for the financially vulnerable: Design and effectiveness
Source: Front Psychol. 2022 Dec 15;13:1090024. doi: 10.3389/fpsyg.2022.1090024 (PMC9798916; doi:10.3389/fpsyg.2022.1090024)
Supplement: Supplementary file 1 [file Data_Sheet_1.pdf]

Supplementary material for:

De Bruijn, E., Antonides, G., and Madern, T. (2022). A behaviorally informed financial education program for the financially vulnerable: Design and effectiveness.

Overview of the developed measurement instruments

Financial skills & knowledge scale*

(1) I know the amount of my fixed costs
(2) I know which letter or email I have to keep and which I can throw away.[1]
(3) I know which insurances I need.
(4) I know where I can get assistance for my financial matters.
(5) I know how to make a budget plan
(6) I know how to keep track of my income and expenses.
(7) I know how to apply for allowances.
(8) I know how to take out insurance.

Keeping-track scale*

(1) I keep track of my expenses using a clear overview.
(2) I keep important documents neatly in a permanent place, such as bills, pay slips, and letters.
(3) I read important letters or email, for example, letters from the bank.
(4) I check how much money I spend and how much money I receive.
(5) I keep track of my financial affairs.[2]

Conscious consumption scale*

(1) Before I buy something, I always consider whether I can afford it.[3]
(2) Before I buy something, I always consider whether I need it.
(3) I do my best to spend little money.

Chronic financial stress scale

How often does it occur, as a consequence of your financial situation or money affairs, that you:

(1) Are physically strained
(2) Face sleep difficulties
(3) Have a headache
(4) Are irritated
(5) Are anxious

Answer scale: never, sometimes, regularly (weekly), often, always (every day).[4]

---

[1] This item was based on: Witvliet, M., Madern, T., and van der Werf, M. (2014). Validatie Mesis©: Verslag van onderzoeken naar interne en concurrente validiteit van het methodisch screeningsinstrument schulddienstverlening, Mesis©. Available at: https://www.mesis.nu.

[2] This item was derived from: OECD (2015). 2015 OECD/INFE toolkit for measuring financial literacy and financial inclusion. Available at: https://www.oecd.org/daf/fin/financial-education/2015_OECD_INFE_Toolkit_Measuring_Financial_Literacy.pdf.

[3] This item was derived from OECD (2016). OECD/INFE international survey of adult financial literacy compentencies. Available at: http://www.oecd.org/finance/oecd-infe-survey-adult-financial-literacy-competencies.htm.

[4] The additional phrases within parentheses were included to create reference points.

Program evaluation

*Usefulness of (aspects of) program**

(1) I found the working materials useful and informative.
(2) I found the homework assignments practical and informative.
(3) I found the course useful and informative.
(4) The course fitted well with my needs.

*Program satisfaction***

(1) Taking everything into account, how satisfied are you with the course?

Perceived improvements in outcomes

*Financial management**

(1) Due to the course, I know more about my financial affairs.
(2) Due to the course, I know better how to properly keep track and manage my financial affairs.
(3) Due to the course, I have a better overview of my financial affairs.
(4) The course has helped me to better manage my money.

*Financial-psychological indicators**

(1) Due to the course, I have more self-confidence to properly manage my financial affairs.
(2) Due to the course, I am more motivated to keep track of my financial affairs.
(3) Due to the course, I can better induce myself to keep track of my financial affairs.

*Implementation tools**

(1) I received many tips during the course to better manage my financial affairs.
(2) Due to the course, I better manage to set small goals for my financial affairs. And I even better manage to achieve those goals.

* Answer scale for all items: 5-point Likert-type scale (1 = *totally disagree* and 5 = *totally agree).*
** Answer scale: 5-point Likert-type scale (1 = *very unsatisfied* and 5 = *very satisfied*).
